# Tonga in Neuralgia

**Published:** 1880-08

**Authors:** 


### TONGA IN NEURALGIA.

Dr. Ringer, in a short article on *tonga*, states the following points: It is a remedy, probably a combination of different barks, in use among the Fiji Islanders. A fluid extract, in drachm-doses three times a day, cured promptly six cases of severe neuralgia, improved a seventh, and failed in the eighth, only because, as Dr. Ringer believes, the preparation had become inert. These cures were produced without toxic symptoms in any case. Sensation, the secretions of the mouth, and the pupil were unaffected; large doses (℥ ss) produced slight drowsiness in one patient.

